# Multi-target antidiabetic and organ-protective effects of a polyherbal ethanol extract in STZ-induced diabetic rats

**DOI:** 10.3389/fphar.2026.1870583

**Published:** 2026-06-30

**Authors:** Mohd Adnan Kausar, Kehkashan Parveen, Sadaf Anwar, Yusuf Saleem Khan, Ayman A. Saleh, Mai Ali Abdelfattah Ahmed, Ehab Adel Awad Abdel Razek, Suhel Parvez, Waseem Ahmad Siddiqui

**Affiliations:** 1 Department of Biochemistry, College of Medicine, University of Ha’il, Hail, Saudi Arabia; 2 Department of Medical Elementology and Toxicology, School of chemical and Life Sciences, Jamia Hamdard, New Delhi, India; 3 Interdisciplinary Biotechnology Unit, Aligarh Muslim University, Aligarh, Uttar Pradesh, India; 4 Department of Anatomy, College of Medicine, University of Ha’il, Hail, Saudi Arabia; 5 Department of Pathology, College of Medicine, University of Ha’il, Hail, Saudi Arabia; 6 Department of Pediatrics, College of Medicine, University of Ha’il, Hail, Saudi Arabia; 7 Department of Sports Science and Physical Activity, College of Education, University of Ha’il, Hail, Saudi Arabia

**Keywords:** diabetes mellitus, hepatic injury, oxidative stress, pancreas, polyherbal extract

## Abstract

**Background:**

In diabetes mellitus conditions, hyperglycemia leads to oxidative stress, inflammation, and β-cell dysfunction, and traditional pharmacotherapy targets only a single pathway, which gives suboptimal outcomes and drug-related problems. A novel, polyherbal ethanol extract (PHE) formulation from *Tinospora cordifolia, Commiphora wightii, Cinnamomum zeylanicum,* and *Paeonia officinalis* was evaluated for antidiabetic and organ-protective effects at different levels for multi-targeting in a streptozotocin (STZ)-induced rat model.

**Methods:**

Male Wistar rats were made diabetic using intraperitoneal administration of streptozotocin (55 mg/kg) and randomly segregated into five groups: Normal, STZ-induced diabetic, STZ + PHE (200 mg/kg/day), Normal + PHE, and STZ + metformin (Met; 300 mg/kg/day). After 8 weeks of treatment, FBG, HbA1c, insulin, lipid profile, proinflammatory cytokines (TNF-α, IL-1β), oxidative stress biomarkers, and liver function markers were determined. Histological examination was performed on liver and pancreatic tissues with H&E staining, along with assessment of pancreatic insulin immunoreactivity. GC–MS analysis was conducted to characterize the phytochemical constituents of PHE.

**Results:**

PHE treatment significantly reduced FBG by 33%, HbA1c by 15%, and restored serum insulin levels (+34%) versus untreated diabetics (p < 0.05). Dyslipidemia was corrected, with LDL-C reduced by 30% and HDL-C increased by 46%. Hepatic ALT and AST levels were also attenuated after PHE treatment. Oxidative stress was alleviated, and TNF-α and IL-1β levels in the liver and pancreas were markedly suppressed. Histology showed preserved hepatocyte integrity and islet morphology, and immunohistochemistry confirmed improved β-cell integrity and enhanced insulin immunoreactivity.

**Conclusion:**

The polyherbal preparation produced impressive control over glycemia, strong antioxidant and anti-inflammatory effects, and protection of liver and pancreatic architecture. These effects translate into its potential as a phytotherapeutic candidate for safely managing diabetes and its complications, warranting further molecular and clinical research.

## 1 Introduction

Diabetes mellitus is a common chronic metabolic disorder associated with persistent hyperglycemia and abnormalities affecting metabolic processes involving proteins, fats, and carbohydrates. The prevalence of diabetes is expected to reach alarming rates by 2030, with projections indicating that over 640 million people globally will have uncontrolled diabetes (Magliano et al., 2021). Current pharmacological agents available for managing diabetes have limited mechanistic coverage, largely failing to address the oxidative-inflammatory milieu that drives diabetic complications, and they pose risks related to long-term polypharmacy (Ansari et al., 2023). Most conventional drugs target either insulin secretion or glucose uptake while ignoring the underlying oxidative-inflammatory environment responsible for diabetic complications. Additionally, prolonged use of these agents may lead to undesirable side effects, reduced compliance, and treatment resistance. This has prompted increasing interest in phytotherapeutic alternatives, especially those with multi-targeted potential (Lakshmi and Ganta, 2023; Sheethal et al., 2023).

Polyherbal formulations have been used for the treatment of chronic diseases such as diabetes in Ayurveda and traditional Chinese medicine systems. Polyherbalism, defined as the combination of two or more herbs for therapeutic use, ensures the synthesis of synergistic therapeutic benefits and minimization of toxicity (Parasuraman et al., 2014). In this work, a polyherbal ethanol extract (PHE) was developed from four medicinal plants: *Tinospora cordifolia* (stem), *Commiphora wightii* (bark), *Cinnamomum zeylanicum* (bark), and *Paeonia officinalis* (root), all of which have individually been reported to exert antidiabetic effects through distinct yet complementary therapeutic actions.

*Tinospora cordifolia* has gained importance for its antidiabetic, immunomodulatory, and hepatoprotective activities. Traditionally used to alleviate symptoms of metabolic dysfunction such as excessive thirst, fatigue, and frequent urination, it has been experimentally documented to lower blood glucose, improve glucose tolerance, and promote β-cell regeneration (Stanely et al., 2000; Mandar et al., 2021). *Commiphora wightii* (Guggul) is a resinous plant used in Ayurvedic formulations for obesity, hyperlipidemia, and metabolic syndrome. Its bark extract has been reported to reduce serum glucose and improve lipid profiles in diabetic rats (Batiha et al., 2023). Alongside antihyperglycemic properties, Guggul exhibits cardioprotective and anti-inflammatory effects relevant to diabetic complication management (Ramesh et al., 2015). *Cinnamomum zeylanicum*, known as true or Ceylon cinnamon, improves insulin sensitivity and glycemic control and has traditionally been used to aid digestion and circulation. Cinnamon has been shown to reduce fasting blood glucose levels and glycosylated hemoglobin in clinical and preclinical settings (Khan et al., 2003; Ranasinghe et al., 2013). It also exhibits antioxidant and hypolipidemic effects beneficial for managing diabetes-associated comorbidities (Silva et al., 2022). *Paeonia officinalis*, the European peony, is traditionally used in European and Chinese medicine for inflammatory, gynecological, and neurological disorders. Recent studies have reported antidiabetic effects of its root extract in animal models, including improved glucose metabolism, reduced oxidative stress, and organ-protective effects in the liver and pancreas (Li et al., 2023; Ou et al., 2025).

Based on the complementary therapeutic benefits of these four botanicals, it was hypothesized that PHE may provide superior antiglycemic and organ-protective effects in diabetic states. Therefore, the aim of this study was to evaluate the antidiabetic, anti-inflammatory, and antioxidant efficacy of PHE in streptozotocin (STZ)-induced diabetic Wistar rats, with a focus on the liver and pancreas. Outcome measures included metabolic indices, inflammatory cytokines, oxidative stress biomarkers, and histopathological changes in these organs.

## 2 Materials and methods

### 2.1 Plant material collection and extract preparation

Stems of *T. cordifolia*, bark of *C. wightii* and *C. zeylanicum*, and roots of *P. officinalis* were procured from the Herbal Garden, Jamia Hamdard. Botanical identification was performed by a qualified taxonomist in the Department of Botany, Jamia Hamdard, New Delhi. Voucher specimens were not formally deposited in a recognized herbarium at the time of the study; therefore, voucher specimen numbers are not available. All plant materials were shade-dried, pulverized, and sieved through mesh size 40. Equal weights of each powdered plant part (1:1:1:1) were mixed. The equal-ratio formulation was chosen in order to support a balanced presence of all four medicinal plants, and to enable potential synergistic interactions among their bioactive constituents. While the individual plant materials may vary in extraction yields and phytochemical composition, an equal-weight ratio was used, to provide a standardized and reproducible formulation for the initial evaluation of the polyherbal extract. Optimization of the individual component ratios based on extraction yield, phytochemical profile, and pharmacological activity was beyond the scope of the present study and will be investigated in future studies. PHE was prepared by Soxhlet extraction of the combined powder (about 500 g) with 70% ethanol (v/v) for 72 h under controlled heating, followed by concentration under reduced pressure at 40 °C and storage at 4 °C in amber vials until analysis (Kausar et al., 2022). Percentage yield was calculated as the ratio of dried extract obtained to the initial weight of plant material used.

### 2.2 Phytochemical characterization and quality control

Phytochemical characterization of PHE was performed using a Shimadzu GC–MS QP2010 Plus equipped with an Rtx-5MS capillary column (30 m × 0.25 mm, 0.25 µm film thickness). Analytical conditions included helium as the carrier gas at 1.0 mL/min (constant flow), splitless injection of 1 µL sample at 250 °C, and oven temperature programmed from 60 °C (held for 2 min) to 280 °C at 10 °C/min (held for 10 min). Electron impact ionization was conducted at 70 eV, with a scan range of m/z 50–600. PHE (100 mg) was dissolved in 1 mL analytical-grade ethanol, filtered through a 0.22 µm membrane, and injected for analysis. Compound identification was performed by comparison of mass spectra with entries in the NIST mass spectral library database. Aliquots from the same analytically characterized extract batch were used throughout the study to ensure experimental consistency and reproducibility. Although GC-MS profiling was performed to characterize the phytochemical composition of the extract, quantitative standardization using specific phytochemical marker compounds was not undertaken and should be considered in future studies.

### 2.3 Experimental animals and housing

Male Wistar rats (180–220 g) were housed at 25 °C and 55 ± 5 percent relative humidity under standard laboratory conditions with a 12 h light/dark cycle. Ethical approval was obtained from the Institutional Animal Ethical Committee, Jamia Hamdard, Delhi (173/CCSEA), and all experiments were conducted in accordance with CCSEA guidelines.

### 2.4 Acute oral toxicity

The safety profile of PHE was assessed according to OECD guideline 423 (Acute Toxic Class Method). Different doses of PHE were administered orally to rats and observed for 30 min immediately after dosing, intermittently for the first 24 h, and daily for 14 days. Parameters recorded included mortality, behavior, autonomic responses, and body weight. No mortality was observed even at the highest dose (2000 mg/kg). Therefore, 200 mg/kg/day (approximately one-tenth of the highest non-toxic dose) was selected for efficacy evaluation. This approach is commonly employed in preclinical pharmacological studies for selecting a safe and pharmacologically active dose (Chopra, 2020; Kausar et al., 2022).

### 2.5 Induction of diabetes

Experimental rats were fasted for 12 h prior to intraperitoneal administration of STZ (55 mg/kg), freshly prepared in ice-cold citrate buffer (pH 4.5). After 72 h, fasting blood glucose (FBG) was measured using a glucometer. Rats with FBG > 250 mg/dL were considered diabetic and included in the study.

### 2.6 Experimental design

Animals were randomized into the following groups (n = 7 each).

- Group I: Normal (saline)
- Group II: STZ-diabetic control
- Group III: STZ + PHE (200 mg/kg)
- Group IV: Normal + PHE (200 mg/kg)
- Group V: STZ + metformin (300 mg/kg).

Treatments were administered orally for eight consecutive weeks.

### 2.7 Biochemical assessments

After 8 weeks, rats were anesthetized with ketamine (80 mg/kg) and xylazine (10 mg/kg) intraperitoneally, and blood was collected by cardiac puncture. Samples were centrifuged at 1000 × g for 10 min at 4 °C, and serum was stored at −20 °C. FBG was measured by the GOD–POD method (Crest Biosystems, India). Serum insulin was quantified using a commercially ultra-sensitive rat ELISA kit (Crystal Chem, USA) according to manufacturer’s instructions. Glycated hemoglobin (HbA1c) was determined using cation-exchange methods (ELK Biotech, China). Lipid parameters were analyzed enzymatically (Agappe Diagnostics, India), with LDL and VLDL calculated using Friedewald’s equation.

### 2.8 Hepatic enzyme activity

Aspartate aminotransferase (AST) and alanine aminotransferase (ALT) activities were assessed following manufacturer protocols (RayBiotech; AST: MA-AST-01; ALT: MA-ALT-01).

### 2.9 Inflammatory cytokines

Serum TNF-α and IL-1β levels were measured using rat-specific ELISA kits (RayBiotech) according to manufacturer instructions.

### 2.10 Oxidative stress markers

Following blood collection, animals were euthanized using a CO_2_ inhalation system. The chamber received CO_2_ at a displacement rate of 30 to 70 percent of chamber volume per minute through a system that included a gas cylinder with a regulator and flow meter as recommended by the AVMA Guidelines for the Euthanasia of Animals (2020). Liver and pancreatic tissues were homogenized in ice-cold phosphate buffer (pH 7.4) and centrifuged at 4000 rpm for 15 min at 4 °C. Supernatants were used to assess malondialdehyde (MDA; thiobarbituric acid method), reduced glutathione (GSH; DTNB method), glutathione-S-transferase (GST; CDNB substrate method), superoxide dismutase (SOD; pyrogallol autoxidation method), and catalase (CAT; H2O2 decomposition method) activities (Marklund and Marklund, 1974; Ohkawa et al., 1979; Habig and Jakoby, 1981; Greenwald, 1985; Gherghel et al., 2005). Protein content was measured for normalization.

### 2.11 Histopathology

Formalin-fixed liver and pancreatic tissues were processed for paraffin embedding, sectioned at 4 μm, and stained with hematoxylin and eosin. After mounting with DPX and xylene washing, slides were examined under a bright-field microscope.

### 2.12 Immunohistochemistry

Pancreatic sections were cryoprotected in 30% sucrose, cut at 30 μm, and sequentially incubated in 3% H_2_O_2_/10% methanol, 10% BSA/PBS-Triton X-100, and anti-insulin antibody (Cat# AF5109; Affinity Biosciences, USA). Sections were treated with biotinylated secondary antibody and avidin–biotin complex, followed by DAB development. Slides were dehydrated, coverslipped, and examined microscopically for β-cell immunoreactivity. Preservation of insulin immunoreactivity was considered indicative of β-cell integrity and functional status.

### 2.13 Statistical analysis

Data were analyzed using SPSS version 23. For multiple group comparisons, one-way ANOVA followed by Tukey–Kramer *post hoc* testing was applied. Results are expressed as mean ± SEM, with significance set at p < 0.05. The use of parametric statistical methods was based on the experimental design, sample size uniformity across groups, and the continuous nature of the measured variables.

## 3 Results

### 3.1 GC–MS profile of PHE

**FIGURE 1:**
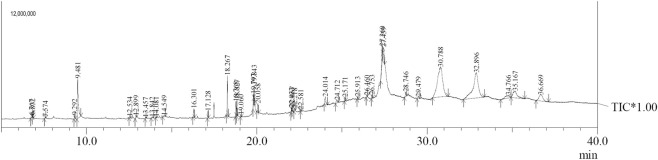
GC–MS chromatogram of PHE demonstrating the presence of different compounds at varying retention times.

GC–MS analysis showed the existence of complex phytochemicals with thirty-nine identifiable constituents, including sterols, fatty acid esters, long-chain alcohols, glycerides, and phenolic derivatives (Supplementary Table S1). Among the notable compounds were ergost-25-ene-3,5,6,12-tetrol (31.21%), tetracosenoic acid methyl ester (26.83%), cinnamaldehyde analogues (6.83%), and cholest-5-en-3-ol (5.55%) (Figure 1, Supplementary Table S1). Many of these metabolites have antioxidant, anti-inflammatory, or insulin-sensitizing activity, which may act in concert in the observed pharmacological effects.

### 3.2 Body weight changes

There was a marked loss in body weight in diabetic animals throughout the observation period (Table 1). Conversely, administration of PHE to diabetic rats reduced weight loss, approximating values to those of the metformin-treated animals. Thus, their metabolic efficiency and nutrient utilization might have improved.

**TABLE 1:** The effect of PHE treatment on body weight of normal and experimental groups.

Groups	Body weight (g)	
	Initial	Final
Normal	185.6 ± 6.2	234.6 ± 6.4
Diabetic	196.6 ± 5.4 (+5.93%)	158.5 ± 5.2^a^ (−32.44%)
Diabetic+PHE	190.7 ± 5.6 (−3.00%)	213.7 ± 4.0^b^ (+34.83%)
Diabetic+Met	188.5 ± 5.5 (−4.12%)	212.6 ± 4.7^b^ (+34.13%)
Normal+PHE	202.4 ± 5.7 (9.05%)	228.6 ± 5.8 (−2.56%)

The study groups’ body weights at initial and final stage. Diabetic group demonstrated considerable changes in body weight in contrast with the normal group. PHE or Met treatment significantly ameliorated body weight when tried to compare with the diabetic group. Values in parentheses indicate percentage increase (+) or decrease (−) as compared with the normal or the Diabetic group. Data are presented as mean ± SEM (n = 7).

^a^ p < 0.05 vs. Normal group;

^b^ p < 0.05 vs. Diabetic group.

### 3.3 Glycemic control and lipid profile

STZ injection was expectedly associated with a marked increase in FBG and HbA1c compared with those of the Normal group (p < 0.05) (Table 2). After 8 weeks of PHE supplementation, a marked decrease in both parameters was recorded when compared with untreated diabetics (p < 0.05); however, no significant difference was observed between the Normal and Normal + PHE groups. Induction of diabetes resulted in lower serum insulin levels (p < 0.05 vs Normal), indicative of β-cell dysfunction. Both the metformin and PHE groups showed significantly increased insulin levels compared to the diabetic group. The PHE group showed an elevation of insulin levels, indicating a probable protective or stimulatory effect of PHE on pancreatic β-cell function. The Normal + PHE group did not display any substantial deviations from the Normal animals.

**TABLE 2:** The effect of PHE treatment on blood glucose, insulin and HbA1c level of normal and experimental groups.

Groups	Blood glucose (mg/dl)	Insulin (ng/ml)	HbA1c (%)
Normal	97.2 ± 3.8	4.1 ± 0.21	5.0 ± 0.15
Diabetic	269.6 ± 6.3^a^ (+177.37%)	2.3 ± 0.14^a^ (−43.90%)	7.2 ± 0.18^a^ (+44%)
Diabetic+PHE	179.8 ± 5.4^b^ (−33.31%)	3.1 ± 0.17^b^ (+34.78%)	6.1 ± 0.21^b^ (−15.28%)
Diabetic+Met	169.6 ± 4.6^b^ (−37.09%)	3.4 ± 0.16^b^ (+47.83%)	6.0 ± 0.19^b^ (−16.67%)
Normal+PHE	89.4 ± 3.1 (−8.02%)	3.9 ± 0.15 (−4.88%)	5.3 ± 0.16 (+6%)

The Diabetic group showed a significant increase in FBG and HbA1c with a significant decrease in insulin compared with the normal group. PHE treatment significantly augmented these parameters in the Diabetic+PHE group compared with the Diabetic group. Values in parentheses indicate percentage increase (+) or decrease (−) as compared with the Normal or the Diabetic group. Data are represented as mean±SEM (n = 7).

^a^ p < 0.05 vs. Normal group;

^b^ p < 0.05 vs. Diabetic group.

Diabetic dyslipidemia was characterized by elevated TG, TC, and LDL-C and decreased HDL-C. PHE treatment normalized the lipid profile by significantly decreasing TG, TC, and LDL-C and increasing HDL-C (p < 0.05). These lipid-lowering effects were comparable to those observed in metformin-treated animals (Table 3).

**TABLE 3:** The effect of PH treatment on lipid profile of normal and experimental groups.

Groups/ Parameters	TC (mg/dl)	TG (mg/dl)	HDL-C (mg/dl)	LDL-C (mg/dl)	VLDL-C (mg/dl)
Normal	145.2 ± 2.9	97.8 ± 1.4	48.4 ± 1.9	77.2 ± 1.3	19.5 ± 1.2
Diabetic	230.9 ± 3.8^a^ (+59.02%)	167.3 ± 3.3^a^ (+71.06%)	26.5 ± 1.2^a^ (−45.25%)	170.9 ± 2.6^a^ (+121.38)	33.4 ± 1.4^a^ (+71.28%)
Diabetic+PHE	184.6 ± 3.3^b^ (−20.05%)	136.8 ± 3.2^b^ (−18.23%)	38.7 ± 1.6^b^ (+46.04%)	118.5 ± 2.2^b^ (−30.66%)	27.4 ± 1.8^b^ (−17.96%)
Diabetic+Met	176.5 ± 3.9^b^ (−23.56%)	129.6 ± 2.6^b^ (−22.53%)	36.4 ± 1.5^b^ (+37.36%)	114.1 ± 2.9^b^ (−33.24%)	25.9 ± 1.6^b^ (−22.46%)
Normal+PHE	146.4 ± 2.8 (+0.83%)	102.5 ± 3.0 (+4.80%)	52.0 ± 1.8 (+7.44%)	73.9 ± 1.7 (−4.27%)	20.5 ± 1.5 (+5.13%)

Data are represented as mean±SEM (n = 7). The Diabetic group showed a significant increase in TC, TG, LDL-C and VLDL-C with a significant decrease in HDL-C compared with the Normal group. PHE treatment significantly augmented these parameters in the Diabetic+PHE group compared with the Diabetic group. Values in parentheses indicate percentage increase (+) or decrease (−) as compared with the Normal or the Diabetic group.

^a^ p < 0.05 vs. Normal group;

^b^ p < 0.05 vs. Diabetic group.

### 3.4 Hepatic enzyme activity

**FIGURE 2:**
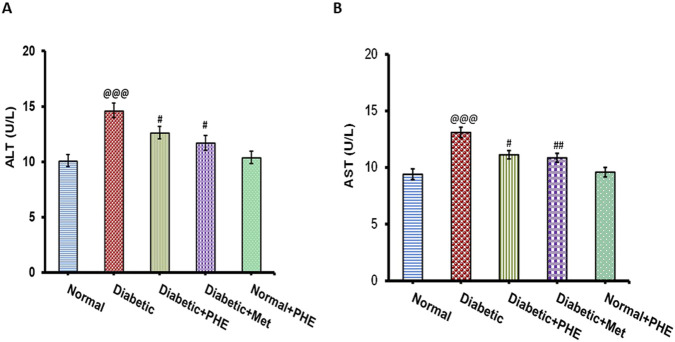
Effects on liver function test: **(A)** ALT and **(B)** AST. There was a significant increase in the ALT and AST levels in diabetic subjects when compared to normal subjects. PHE brought down the ALT and AST levels slightly in Diabetic+PHE in comparison with Diabetics. Values are expressed as mean ± SEM (*n* = 7). *p* values: ^@@@^<0.001; ^@@^<0.01; ^@^<0.05, vs Normal group; *p* values: ^###^<0.001; ^##^<0.01; ^#^<0.05, vs Diabetic group.

Serum ALT and AST were elevated in diabetic rats and are indicators of hepatic injury. PHE treatment decreased the levels of these enzymes, thereby supporting hepatoprotective effects (Figure 2).

### 3.5 Inflammatory cytokines

**FIGURE 3:**
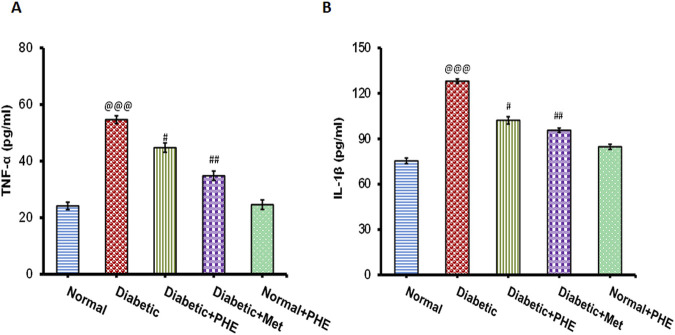
Effects of PHE on proinflammatory cytokines **(A)** TNF-α and **(B)** IL-1β protein expression. The Diabetic group showed a significant increase in TNF-α and IL-1β levels compared to the Normal group. Administration of PHE significantly attenuated the activity of these markers in the Diabetic + PHE group compared to the Diabetic group. Values are expressed as mean ± SEM (*n* = 7). *p* values: ^@@@^<0.001; ^@@^<0.01; ^@^<0.05, vs Normal group; *p* values: ^###^<0.001; ^##^<0.01; ^#^<0.05, vs Diabetic group.

Cytokines TNF-α and IL-1β were highly elevated in the serum of diabetic rats. PHE treatment decreased these cytokines in the diabetic treated group, demonstrating its anti-inflammatory activity (Figure 3).

### 3.6 Oxidative stress parameters

**FIGURE 4:**
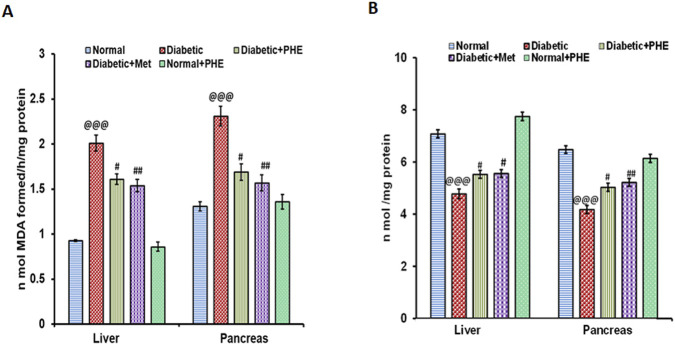
**(A)** Effect of PHE supplementation on MDA levels. High MDA levels were noted in the Diabetic group compared with Normal rats. PHE supplementation significantly reduced MDA levels in the Diabetic + PHE group compared to the Diabetic group. **(B)** Effect of PHE supplementation on GSH levels. The Diabetic group showed a significant reduction in GSH content compared to the Normal group, whereas PHE treatment restored GSH content. Values are expressed as mean ± SEM (*n* = 7). *p* values: ^@@@^<0.001; ^@@^<0.01; ^@^<0.05, vs Normal group; *p* values: ^###^<0.001; ^##^<0.01; ^#^<0.05, vs Diabetic group.

**FIGURE 5:**
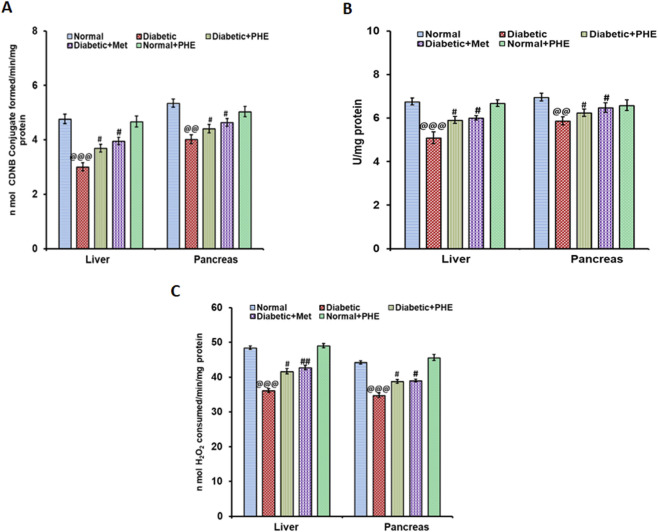
The Diabetic group showed significant alterations in the activities of antioxidant enzymes (GST, SOD, and CAT) compared to the Normal group **(A–C)** Administration of PHE significantly attenuated these alterations in the Diabetic + PHE group compared to the Diabetic group. Values are expressed as mean ± SEM (*n* = 7). *p* values: ^@@@^<0.001; ^@@^<0.01; ^@^<0.05, vs Normal group; *p* values: ^###^<0.001; ^##^<0.01; ^#^<0.05, vs Diabetic group.

Diabetic liver and pancreatic homogenates showed increased levels of MDA and decreased activities of GSH, SOD, and CAT (Figures 4, 5, respectively). Notably, PHE treatment significantly decreased MDA content and restored the activities of antioxidant enzymes toward Normal values (p < 0.05). The Normal and Normal + PHE groups did not show any significant differences.

### 3.7 Histopathology

**FIGURE 6:**
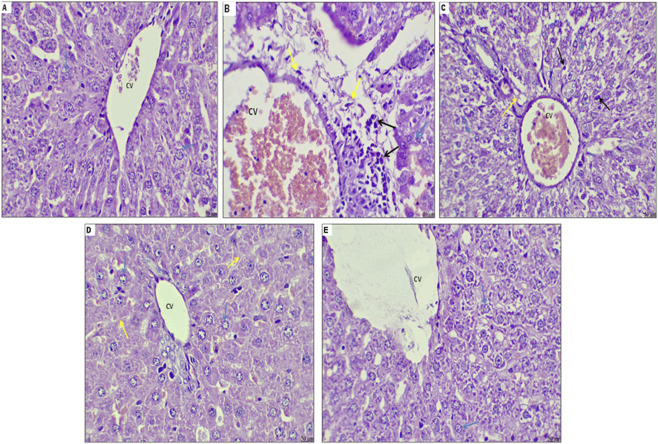
Histological sections of liver tissues. **(A)** Section showing the normal histological appearance of liver tissue in the Normal group. No evidence of cellular disintegration, pyknosis, central vein congestion (CV), or apoptosis was observed. The section also shows normal, trabecular-arranged hepatocytes that are polygonal in shape (blue arrow), with clear cytoplasm and normal round or oval nuclei. No fatty changes, inflammatory infiltrates, or central vein congestion were observed. **(B)** Section from the Diabetic group showing significantly damaged hepatocytes (blue arrow). High cellular density, dense cytoplasm, and significant fatty changes (yellow arrow) were observed. Pyknosis (black arrow), central vein congestion, and inflammatory infiltrates were also present, indicating severe hepatic toxicity. **(C)** Liver section from the PHE-treated group showing mild to moderate histological damage. Mild congestion was observed. The section shows mildly damaged hepatocytes (blue arrow), no vacuolation, mild pyknosis (black arrow), moderate fatty changes (yellow arrow), and moderate inflammatory infiltrates. **(D)** Section from the Met-treated group showing minimal histological damage to liver tissue. No congestion was observed. The section shows mildly damaged hepatocytes (blue arrow), no vacuolation, and mild fatty changes (yellow arrow). **(E)** Section showing the normal histological appearance of liver tissue in the Normal + PHE group. No evidence of cellular disintegration, pyknosis, central vein congestion (CV), or apoptosis was observed. Normal trabecular-arranged polygonal hepatocytes (blue arrow) with clear cytoplasm and normal nuclei were seen, with no fatty changes or inflammatory infiltrates (H&E, 400×).

Sections of the liver from diabetic rats revealed degenerative changes, including hepatocellular necrosis, dilation of the sinusoids, and infiltration of inflammatory cells. Liver sections from PHE-treated diabetic rats were characterized by preserved lobular architecture, reduction in inflammatory infiltrates, and evidence of hepatocyte regeneration (Figure 6).

**FIGURE 7:**
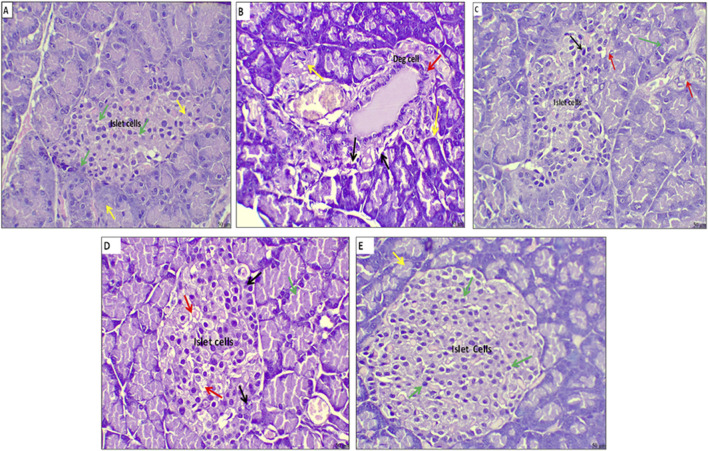
Histopathology of the pancreas. **(A)** Normal group showing normal histological attributes of pancreatic tissue. The islet of Langerhans appears normal, with no evidence of cellular disintegration, pyknosis, or atrophic changes in β-cells (green arrow). Normal pyramidal acidophilic pancreatic acini were observed (yellow arrow). No congestion or hemorrhage was found. **(B)** Diabetic group showing significantly distorted pancreatic lobules and cellular disintegration in the islet of Langerhans (red arrow). Marked pyknosis was observed in β-cells (black arrow). Pancreatic acini were atrophic and significantly disintegrated (yellow arrow). **(C)** PHE-treated sections showing mildly damaged pancreatic lobules and cellular disintegration in the islet of Langerhans (red arrow). Healthy β-cells are indicated by green arrows. Mild pyknosis was observed in β-cells (black arrow). No congestion or hemorrhage was found. **(D)** Met-treated pancreatic sections showing mild to moderate damage to pancreatic lobules and cellular disintegration in the islet of Langerhans (red arrow). Healthy β-cells are indicated by green arrows. Moderate pyknosis was observed in β-cells (black arrow). No congestion or hemorrhage was found. **(E)** Normal rats administered PHE showing normal histological attributes of pancreatic tissue. The islet of Langerhans appears normal, with no evidence of cellular disintegration, pyknosis, or atrophic changes in β-cells (green arrow). Normal pyramidal acidophilic pancreatic acini were observed (yellow arrow). No congestion or hemorrhage was found. Magnification 400×; scale bar 50 µm.

Pancreatic histology in the diabetic control group showed shrinking of islets and depletion of β-cell tissue. PHE improved islet morphology by maintaining β-cell density and reducing degenerative changes, similar to the improvements seen in metformin-treated animals (Figure 7).

### 3.8 Immunohistochemical findings

**FIGURE 8:**
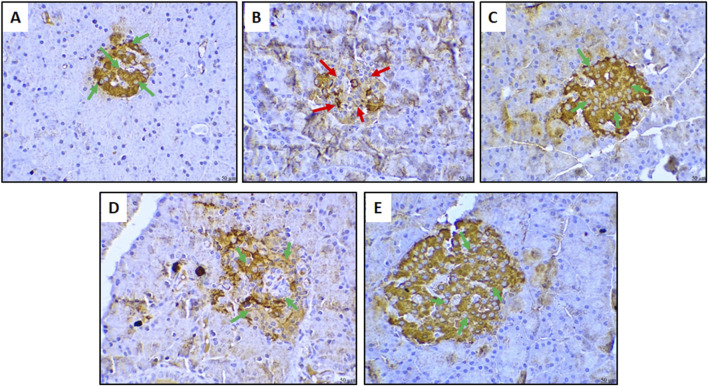
Immunohistochemistry of the pancreas. **(A)** Photomicrograph showing immunohistochemical analysis of insulin in the Normal control group. Strong insulin expression was observed and considered insulin-positive β-cells (green arrow). **(B)** Photomicrograph showing immunohistochemical analysis of insulin in the Diabetic group. Significantly reduced or minimal insulin expression was observed and considered insulin-negative β-cells (red arrow). **(C)** Photomicrograph showing immunohistochemical analysis of insulin in the Diabetic group treated with PHE. Strong insulin expression was observed and considered insulin-positive β-cells (green arrow). **(D)** Photomicrograph showing immunohistochemical analysis of insulin in the Diabetic group treated with the experimental drug. Moderately increased insulin expression was observed and considered insulin-positive β-cells (green arrow). **(E)** Photomicrograph showing immunohistochemical analysis of insulin in the Normal + PHE group. Strong insulin expression was observed and considered insulin-positive β-cells (green arrow). Magnification 400×; scale bar 50 µm.

In normal rats, insulin-positive β-cells formed a dense central core within the islets. Diabetic controls showed a marked reduction in insulin immunoreactivity and β-cell numbers (Figure 8). PHE treatment increased both the intensity of insulin staining and the number of immunoreactive β-cells, indicative of preserved β-cell morphology and function.

## 4 Discussion

The present study provides in-depth mechanistic insight into the therapeutic effects of a PHE of *T. cordifolia, C. wightii, C. zeylanicum*, and *P. officinalis* in the amelioration of diabetic complications in STZ-induced diabetic rats, with particular reference to the biochemical, histological, and physiological benefits brought about by individual but synergistic pharmacological actions. The following discussion refers to the mechanistic relevance of each herb by relating the pathological parameters assessed in this study to results obtained in previous literature.

GC–MS investigation revealed bioactive constituents, including sterols, fatty acid esters, alcohols, aldehydes, and glycerides. The predominant components, ergost-25-ene-3,5,6,12-tetrol and tetracosenoic acid methyl ester, have established antioxidant and insulinotropic activities (Shilpa et al., 2009; Dong et al., 2019). Cinnamaldehyde derivatives promote insulin receptor phosphorylation and block inflammatory cascades through inhibition of JNK and MAPK signaling, aligning with the current findings of decreased cytokine expression and enhanced islet morphology (Waraich et al., 2017; Fatima et al., 2024). Altogether, the phytochemical profile revealed through GC–MS substantiates the multitargeted therapeutic potential of PHE. This comprehensive profile provides a rational basis for the formulation’s effectiveness and scope for future molecular docking and bioavailability studies.

Body weight loss is common in diabetic rats due to increased catabolism. Body weight normalization observed in the treatment group indicates improved nutrient utilization and metabolic efficiency. This effect was probably due to enhanced insulin sensitivity and glucose uptake in peripheral tissues mediated by *T. cordifolia* and *C. zeylanicum* (Mishra et al., 2023; Moridpour et al., 2024). Anabolic metabolism was further improved by decreased inflammation and enhanced lipid handling effects of *C. wightii* (Gorantla et al., 2021).

Decreased levels of FBG and glycated hemoglobin (HbA1c), along with increased insulin levels in PHE-fed rats, could mechanistically be related to activation of pathways such as AMPK, GLUT4 translocation, inhibition of NF-κB, and regulation of IRS-1/Akt signaling through the combined actions of all four herbs (Anand et al., 2010; Mishra et al., 2011; Ma et al., 2017).

Diabetic rats supplemented with PHE showed remarkable improvement in dyslipidemia. Additionally, cholesterol and triglyceride levels were regulated through antagonism of the farnesoid X receptor (FXR) by *C. wightii*, thereby inhibiting hepatic lipogenesis and promoting cholesterol efflux (Urizar and Moore, 2003). *T. cordifolia* has been reported to reduce LDL-C and raise HDL-C (Patil et al., 2011; Shirolkar et al., 2020). *C. zeylanicum* improved lipid metabolism by regulating SREBP-1c expression and reducing triglyceride accumulation (Sheng et al., 2008). *P. officinalis* exhibited hypolipidemic activity through anti-inflammatory actions that indirectly impact lipid homeostasis (Liu et al., 2022).

The assessment of ALT and AST level is one of the most common and popular measures for evaluating hepatocellular necrosis, hepatic injury, membrane disruption, and leakage (Kaplan, 1993; Alabi and Bakare, 2014). The increased activities of serum AST and ALT observed in diabetic rats suggest chronic hepatocellular damage, possibly due to membrane damage or leakage. PHE effectively attenuated these increased levels of AST and ALT due to effective hepatoprotective action of the individual herb present in the extract, corroborated with previous studies (Ahmad and Tabassum, 2013; Asl et al., 2014; Bajaj et al., 2024).

Chronic inflammation exacerbates diabetes, with pronounced effects on hepatic and pancreatic tissues. The present study demonstrated attenuation of pro-inflammatory responses through reductions in TNF-α and IL-1β levels in the PHE-treated group. *C. wightii* emerged as a major contributor, with guggulsterone inhibiting NF-κB translocation and cytokine production (Yamada and Sugimoto, 2016). The anti-inflammatory properties of *T. cordifolia* involve inhibition of macrophage activation via downregulation of iNOS and COX-2 (Singh et al., 2003; Nair et al., 2004). *C. zeylanicum* interrupts inflammatory cascades through inhibition of MAPK and JNK pathways mediated by cinnamaldehyde (Rao and Gan, 2014; Sankaranarayanan et al., 2024). *P. officinalis* demonstrated immunomodulatory activity by suppressing cytokine synthesis through inhibition of TLR4 signaling and reduction of macrophage infiltration (Shao et al., 2019).

Oxidative stress is the basis for tissue injury in diabetes. PHE treatment resulted in significant reductions in both hepatic and pancreatic levels of MDA, accompanied by increased activities of SOD, CAT, and GSH. *T. cordifolia* significantly improved antioxidant enzyme activities due to its high polyphenol and alkaloid content, which directly scavenges reactive oxygen species (ROS) (Ilaiyaraja and Khanum, 2011; Shirolkar et al., 2020). *C. wightii* contributes through guggulsterone-mediated upregulation of Nrf2 signaling, enhancing endogenous antioxidant defenses (Gupta et al., 2022). *C. zeylanicum* contains polyphenolic compounds that inhibit lipid peroxidation and stabilize mitochondrial function (Weerasekera et al., 2021). Rich in paeoniflorin, *P. officinalis* acts as an effective free radical scavenger and protects tissues from oxidative damage by inhibiting ROS-generating enzymes (Lu et al., 2024).

Histological observations confirmed improved hepatic lobular architecture and reformed pancreatic islets in PHE-treated rats. *T. cordifolia* and *P. officinalis* exerted their effects through antioxidant, anti-apoptotic, and anti-inflammatory mechanisms (Liu et al., 2019; Mansouri et al., 2025). *C. wightii* reduced lymphocytic infiltration and hepatocyte ballooning via its anti-inflammatory action (Yamada and Sugimoto, 2016). *C. zeylanicum* enhanced insulin secretion while preventing β-cell death, thereby improving islet density and structural restoration (Waraich et al., 2017).

Immunohistochemical assays further indicated that treatment with PHE preserved the architecture of pancreatic β-cells and insulin expression in STZ-induced diabetic rats. Diabetic controls exhibited markedly reduced insulin immunoreactivity, consistent with STZ-induced β-cell cytotoxicity and islet disruption. In contrast, the PHE-treated group showed strong insulin positivity within islets of Langerhans, suggesting enhanced β-cell survival and partial regeneration. Mechanistically, this protective effect may result from combined actions of the constituent herbs. *T. cordifolia* may promote β-cell neogenesis and upregulate insulin gene expression via modulation of GLUT4 and PDX-1 pathways (Sangeetha et al., 2013; Damame et al., 2022). *C. wightii*, through guggulsterone, inhibits NF-κB activation and cytokine-mediated β-cell apoptosis (Shishodia and Aggarwal, 2004). *C. zeylanicum* enhances β-cell function and glucose-stimulated insulin secretion through activation of AMPK and insulin receptor signaling (Beheshti et al., 2025). Preservation of β-cell integrity may also be mediated by *P. officinalis*, whose major component paeoniflorin downregulates JNK phosphorylation and inhibits MAPK-mediated pro-apoptotic signaling (Mishra et al., 2023). Collectively, these mechanisms likely contributed to improved insulin immunoreactivity, restored islet architecture, reduced hyperglycemia, and reestablished metabolic homeostasis.

A limitation of the present study is the evaluation of only a single therapeutic dose of PHE. Although the selected dose was based on acute toxicity findings and demonstrated significant biological activity, dose-response studies are required to determine the minimum effective dose, optimal therapeutic dose, therapeutic window, and safety margins.

## 5 Conclusion

In conclusion, the polyherbal formulation exerted therapeutic benefits on glucose and lipid metabolism, oxidative stress, and inflammatory cytokine suppression through synergistic modulation of multiple pathogenic mechanisms involved in diabetes. These preliminary findings strongly support the need for targeted molecular validation and early-phase clinical trials.

## Data Availability

The original contributions presented in the study are included in the article/Supplementary Material, further inquiries can be directed to the corresponding authors.

## Supplementary material

SUPPLEMENTARY TABLE S1 GC–MS analysis of the polyherbal ethanol extract (PHE) showing the detected compounds, retention times, peak areas, and relative abundances.
