# Supplementary material for: Multi-target antidiabetic and organ-protective effects of a polyherbal ethanol extract in STZ-induced diabetic rats
Source: Front Pharmacol. 2026 Jun 30;17:1870583. doi: 10.3389/fphar.2026.1870583 (PMC13364602; doi:10.3389/fphar.2026.1870583)
Supplement: Supplementary file 1 [file Table1.pdf]

Supplementary Table S1: GC–MS analysis of the polyherbal ethanol extract (PHE) showing the detected compounds, retention times, peak areas, and relative abundances.

Peak Report TIC

Peak#	R.Time	Area	Area%	Name
1	6.767	95141	0.26	OXETANE, 2-PROPYL-
2	6.832	39768	0.11	4-Isopropyl-1-methylcyclohex-2-enol
3	7.574	48130	0.13	4H-Pyran-4-one, 2,3-dihydro-3,5-dihydroxy-6-methyl-
4	9.292	127154	0.35	Benzene, (1-methoxyethyl)-
5	9.481	2463653	6.83	2-Propenal, 3-phenyl-
6	12.534	77042	0.21	.BETA.-D-GLUCOPYRANOSE, 1,6-ANHYDRO-
7	12.899	93146	0.26	2-Propenal, 3-(2-methoxyphenyl)-
8	13.457	33185	0.09	BUTANOIC ACID, ANHYDRIDE
9	13.842	58768	0.16	Silane, (2-ethoxycyclohexyl)trimethyl-, cis-
10	14.081	24743	0.07	3,7-Cycloundecadien-1-ol, 1,5,5,8-tetramethyl-
11	14.549	112215	0.31	MOME INOSITOL
12	16.301	286081	0.79	TETRADECANOIC ACID, TRIMETHYLSILYL ESTER
13	17.128	193099	0.54	Hexadecanoic acid, methyl ester
14	18.267	1234666	3.42	Carbonic acid, monoamide, N-octadecyl-, decyl ester
15	18.768	409061	1.13	9,12-OCTADECADIENOIC ACID (Z,Z)-, METHYL ESTI
16	18.827	418360	1.16	9-Octadecenoic acid, methyl ester, (E)-
17	19.060	68698	0.19	Hexadecanoic acid, 15-methyl-, methyl ester
18	19.797	249384	0.69	(Z)-1,3-Dimethoxypropan-2-yl octadec-11-enoate
19	19.843	470895	1.30	MYRISTOLEIC ACID 1TMS
20	20.058	370101	1.03	1H-INDENE, 2-HEXADECYL-2,3-DIHYDRO-
21	22.033	74849	0.21	1,8,11-Heptadecatriene, (Z,Z)-
22	22.067	81153	0.22	Glycidyl oleate
23	22.218	101817	0.28	Behenic alcohol
24	22.581	47805	0.13	5.ALPHA.-CHOLEST-3-ENE, 2.ALPHA.-METHYL-
25	24.014	541306	1.50	2-Oleoylglycerol, 2TMS derivative
26	24.712	246188	0.68	Cholesta-7,9(11)-dien-3-ol, 4,4-dimethyl-, (3.beta.,5.alpha.)
27	25.171	124354	0.34	TETRACONTANE
28	25.913	85431	0.24	TETRATETRACONTANE
29	26.460	89126	0.25	Eicosanoic acid, 2-[(1-oxohexadecyl)oxy]-1-[[(1-oxohexad
30	26.753	146323	0.41	Hexatriacontane
31	27.360	1060726	2.94	Adipic acid, eicosyl 4-heptyl ester
32	27.439	622725	1.73	TETRACOSENOIC ACID, 2-[(TRIMETHYLSILYL)OXY
33	28.746	277533	0.77	16-HENTRIACONTANONE
34	29.479	167317	0.46	RETINOL
35	30.788	11261559	31.21	Ergost-25-ene-3,5,6,12-tetrol, (3.beta.,5.alpha.,6.beta.,12.be
36	32.896	9681671	26.83	TETRACOSENOIC ACID, 2-[(TRIMETHYLSILYL)OXY
37	34.766	803506	2.23	Pentadecafluorooctanoic acid, dec-2-yl ester
38	35.167	2002258	5.55	CHOLEST-5-EN-3-OL (3.BETA.)-
39	36.669	1796780	4.98	Cholesta-5,20-dien-3-ol, (3.beta.)-
		36085717	100.00	
